# Phosphorus status and adsorption characteristics of perennial vegetable-cultivated soils in South China

**DOI:** 10.1371/journal.pone.0264189

**Published:** 2022-04-14

**Authors:** Jianfeng Ning, Jianwu Yao, Ronghui Wang, Yichun Li, Mengjun Li, Jian Shen, Yong Chen, Shijian Zhu, Siyuan Wang, Jiling Luo, Tong Li, Ruikun Zeng, Shaoying Ai

**Affiliations:** Institute of Agricultural Resources and Environment, Guangdong Academy of Agricultural Sciences, Key Laboratory of Plant Nutrition and Fertilizer in South Region, Ministry of Agriculture and Rural Affairs, Guangdong Key Laboratory of Nutrient Cycling and Farmland Conservation, Guangzhou, PR China; Ghazi University, PAKISTAN

## Abstract

Phosphorus (P) is an essential element for crop production and a key source of nonpoint pollution in agroecosystems. In this study, we sought to analyze P levels and the factors affecting soil P availability, via P adsorption, in a typical field system that is characterized by the year-round cultivation of vegetables. A total of 190 sites were sampled from vegetable fields in Guangdong Province, South China. Within the research area, average concentrations of 124.49 mg P kg^-1^ and 1.55 g P kg^-1^ were recorded for available P (AP) and total P (TP), respectively, which are 8.53- and 1.78-fold higher, respectively, than the corresponding values recorded in 1980. The determined P adsorption maximum (Q_m_) averaged at 488.38 mg kg^-1^, which represents a reduction of 16% compared to the values obtained four decades ago. Accumulations of both TP and AP were found to be negatively correlated with the soil’s maximum adsorption buffering capacity (MBC), although no significant correlations with the soil binding energies (k) and Q_m_, were seen. However, soil pH was found to be significantly correlated with k and Q_m_. Furthermore, both free Mn oxides (Mn_d_) and silt concentrations in the soil were found to contribute to explaining the variations in Q_m_. Collectively, the findings of this study provide evidence to indicate that there has been an excessive accumulation of P in the perennial vegetable fields of Guangdong Province over the past four decades, which may have had negative effects on the P supply potential of the soil by reducing the maximum adsorption buffering capacity.

## 1. Introduction

Phosphorus (P) is a limiting factor in nutrient cycling and a key element controlling primary production in agroecosystems. Accordingly, large amounts of chemical P fertilizers have been produced and applied to ensure high crop yields [1, 2]. Given that the P fertilizer applied to soil is strongly adsorbed by clay minerals, approximately 80% of added P accumulates within the soil [3, 4]. In China, it has been estimated that from 1980 to 2007, an average of 242 kg P ha^-1^ accumulated in soil accompanied by an increase in Olsen P from 7.4 to 24.7 mg kg^-1^ [5], which corresponds to an annual accumulation rate of 0.64 mg P kg^-1^. In Guangdong Province, South China, the application of chemical fertilizer on farmland is 1.39 times higher than that at the national level [6]. Notably, vegetable production is often characterized by intensive cropping rotations, farmers usually apply an excessive phosphorus fertilizers to sustain the yields due to the complex P dynamics and poor P uptake capability of vegetables [7–10]. Currently, however, there is insufficient detailed information on soil P status, particularly with respect to the intensively managed vegetable cropping systems in Guangdong Province.

In most of the current management strategies, soil P testing for agronomic purposes is often the only available information about soil P levels [11]. In this context, Olsen’s bicarbonate extractable P (Olsen P) is routinely used to estimate soil P availability for plant uptake, as the Olsen P test is highly dependent on P sorption capacity [12, 13]. Al oxides, Fe (hydr) oxides, and clay minerals [14, 15] are considered to be the major P sorbents in soil and their roles in soil P adsorption have been well-documented [16–20]. Other properties, such as soil parent material [21], pH [22], and texture [23] have also been associated with soil P sorption capacities, along with exchangeable Ca and Mg, and organic matter content [24, 25]. Furthermore, anthropogenic activity, notably fertilization, has proven to be an important factor influencing soil P adsorption capacity, maximum buffering capacity, and P biological availability [26–28]. To date, however, there have been very few studies that have investigated the relationship between soil P status and adsorption characteristics.

The objectives of this study were (i) to investigate P status in the soil of intensively managed vegetable fields, and (ii) to examine P adsorption characteristics and their relationships with soil P status, as well as other soil properties, in these heavily fertilized agricultural systems. We anticipate that the findings of this study will contribute to developing appropriate P management strategies for vegetable production and environmental protection in P-enriched soils.

## 2. Materials and methods

### 2.1 Study site

**Fig 1:**
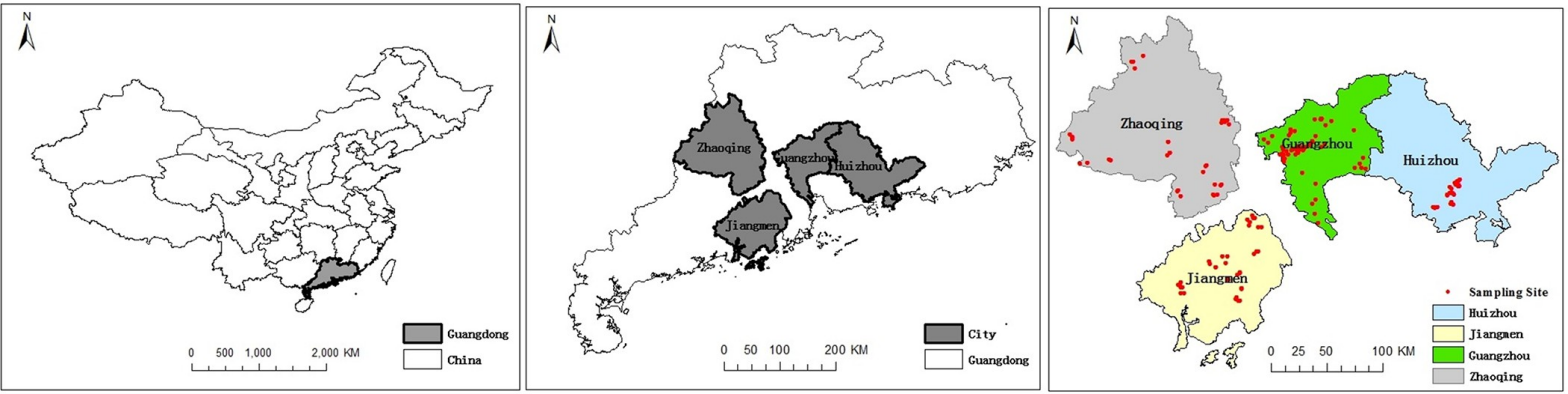
Map of study area with location of sampling sites (A, map of China; B, map of Guangdong province; C, map of sampling sites).

This study was conducted in some areas of the Pearl River Delta (Fig 1), Guangdong province, south China. The study area is in the suburb of the city of Guangzhou (E113°2′15"-113°51′16.7", N 22°44′12"-23°34′54.6"), Huizhou (E113°2′15"-113°51′16.7", N22°44′12"-23°34′54.6"), Jiangmen (E113°2′15"-113°51′16.7", N22°44′12"-23°34′54.6") and Zhaoqing (E113°2′15"-113°51′16.7", N22°44′12"-23°34′54.6"), with a subtropical monsoon climate. Mean annual temperature is 22.4°C and mean annual precipitation 1898.5 mm (1980–2019), with 80% of precipitation occurs between April and September. Based on the Great Groups in Chinese Soil Taxonomy [29], the soils in the research area are classified as latosolic red soils, paddy soils, fluvi-aquic soils and some other soil types (i.e. red soil, purpholish soils, yellow soils, and so on). Latosolic red soils, corresponding to Ultisols [30], are the dominant soil type across the study area, covering 42115.52 km^2^ or approximately 97.5% of the total soil area [31]. Perennial vegetable-cultivated field is a major agricultural system in the study area, various of vegetables such as leafy vegetables, melon vegetables, rhizome vegetables as well as solanaceous fruit vegetables were planted with random rotation annually. Most of sampling sites in the research area have been cultivated vegetables all year round for more than 5 years.

### 2.2 Soil sampling

Soil samples were collected from 81, 37, 36 and 36 vegetable fields in Guangzhou, Huizhou, Zhaoqing and Jiangmen (Fig 1), respectively, during the February to May, 2019. There were 190 soil samples totally collected in the research area. In each sampling site, one field was selected for soil sample collection. Nine soil cores (diameter = 3.0 cm) in each field were taken from the surface soil layer (0–20 cm), avoiding the area of fertilizer or ridges. The moist soil cores from each field were mixed to form a field composite samples which were sieved to a 2 mm-mesh to remove plant residues and visible rocks. Soil samples were air-dried and then stored at room temperature before analysis.

### 2.3 Soil analysis

All soil samples were used for TP and AP analysis. Eleven of 190 samples were selected randomly for phosphorus adsorption isotherm experiments and measurement of soil properties (Table 1).

**Table 1:** General characteristics of the eleven samples for P adsorption isotherm experiment and soil properties analysis.

Soil samples	Geographical coordinates		Vegetable crops
S1	113° 19′11.9" E	23° 21′28" N	Leafy vegetables
S2	113° 26′57.3" E	22° 55′41.7" N	Rotation of leafy vegetables, melon vegetables and bean vegetables
S3	113° 29′55" E	23° 21′00" N	Rotation of bean vegetables, melon vegetables and leafy vegetables
S4	113° 28′23" E	22° 44′12" N	Melon vegetables
S5	113° 31′58" E	23° 31′40" N	Rotation of leafy vegetables and melon vegetables
S6	113° 06′18" E	23° 26′15" N	Rotation of melon vegetables and bean vegetables
S7	113° 51′17" E	23° 10′43" N	Rotation of melon vegetables and bean vegetables
S8	112° 44′32.7" E	23° 16′46" N	Leafy vegetables
S9	112° 03′36.7" E	24° 4′58.9" N	Leafy vegetables
S10	112° 37′42.2" E	22° 57′50" N	Melon vegetables
S11	114° 32′44.2" E	22° 54′15.6" N	Melon vegetables

According to the methods described by Lu (2000) [32], soil pH was measured in a mixture (soil:water 1:2.5) using glass electrode, organic C with potassium bichromate-sulfuric acid method, TP was extracted by digestion with HF-HClO_4_ and determined with molybdenum-blue colorimetry, AP with Olsen method, cation exchange capacity (CEC) with ammonium acetate method and the content of sand, silt and clay was measured with hydrometer method. Free Fe-Al-Mn oxides were extracted with the sodium dithionite-sodium citrate-bicarbonate (DCB) method [33], while amorphous Fe-Al oxides were extracted with ammonium oxalate (pH 3.0) [34]. Fe, Al and Mn in the extracts were determined by using inductively coupled plasma-mass spectrometer (Agilent 7800 ICP-MS, USA).

### 2.4 Phosphorus adsorption

#### 2.4.1 Batch experiment

Phosphorus adsorption was evaluated in triplicate 1.00-g soil samples that were equilibrated in 100 ml centrifuge tubes with 20 ml of CaCl_2_ solution (0.01 mol L^-1^) containing 0, 10, 20, 40, 60, 100 and 150 mg P L^-1^. Three drops of chloroform were added to each tube to inhibit microbial activity. And then, the tubes were shaken for 24 h on an end-over-end shaker at 180 oscillations per min, and centrifuged at 4000 rpm for 10 min. The contents of each tube were then passed through a 0.45 μm membrane filter, and the P concentration in the solution was measured colorimetrically using the molybdate blue method [35].

#### 2.4.2 Modeling of P adsorption

Phosphorus adsorption isotherms were determined with the Langmuir equation (Eq (1)) [36].

$$\frac{C}{Q}=\frac{C}{Q_{m}}+\frac{1}{kQ_{m}}$$ (1)

where Q (mg kg^-1^) is the amount of P adsorbed to the soil at the equilibrium P concentration C (mg L^-1^), Q_m_ (mg kg^-1^) equals P adsorption maximum, and k (L mg^-1^) is a constant related to the binding energy. The maximum adsorption buffering capacity (MBC, L kg^-1^) [37] was expressed as:

$$MBC=k\times Q_{m}$$ (2)

The equilibrium phosphorus concentration at zero adsorption (EPC_0_) indicates the ability of the soil to hold phosphorus [38]. EPC_0_ was calculated with the following formula:

$${EPC}_{0}=\frac{Q_{0}}{K(Q_{m}-Q_{0})}$$ (3)

where Q_0_ is the P concentration in solution when the exogenous P concentration is 0 mg L^−1^.

### 2.5 Statistical analyses

Data were expressed as means of two or three replicates. Correlation and the multiple stepwise linear regression were performed by IBM SPSS statistics versions 17.0. All figures were created using OriginPro 2020 (OriginLab Corporation., Northampton, MA, USA).

## 3. Results

### 3.1 Soil P concentrations

**Fig 2:**
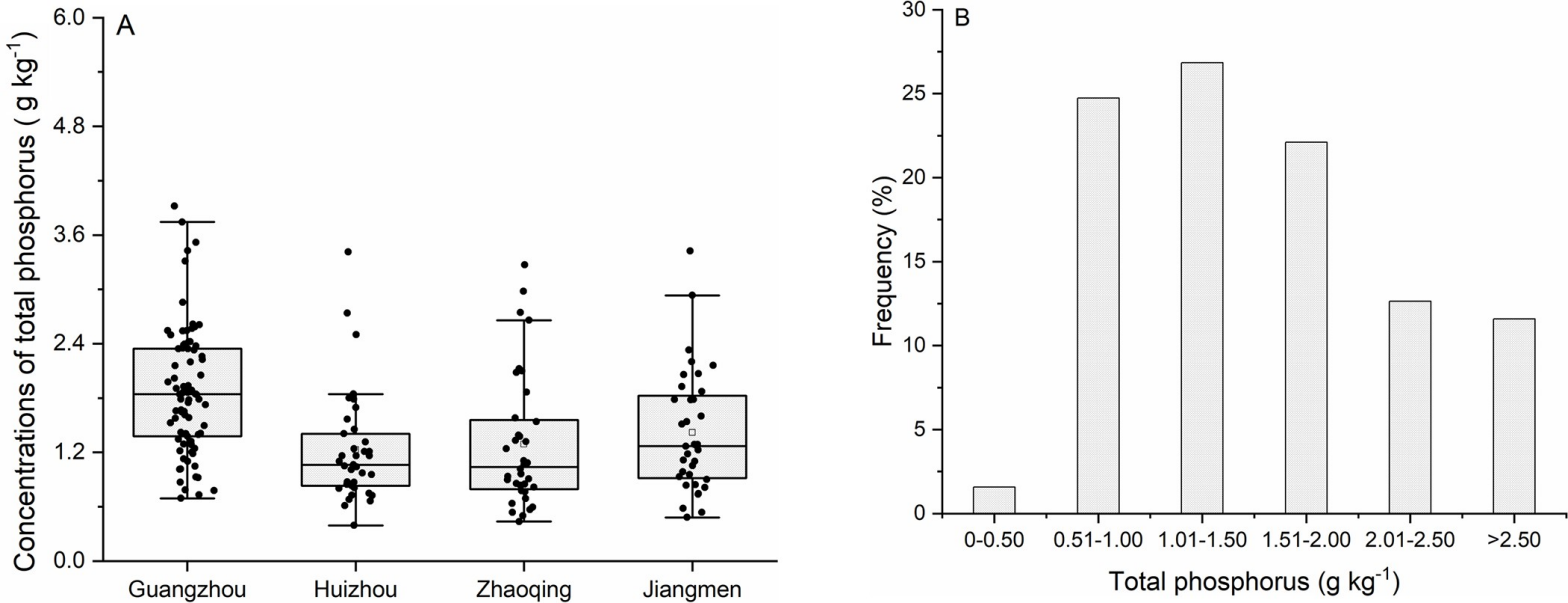
Concentrations (A) and frequency distribution (B) of total phosphorus in studied soils.

**Fig 3:**
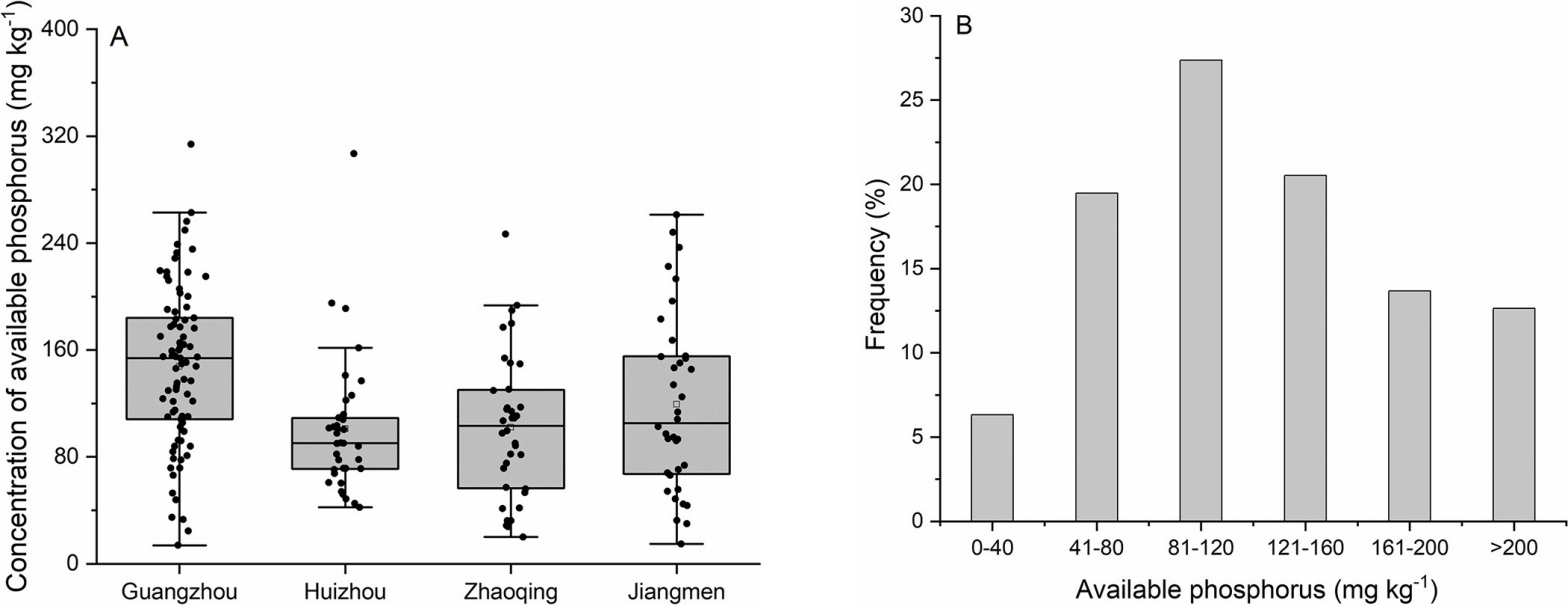
Concentrations (A) and frequency distribution (B) of available phosphorus in studied soils.

In the fields surveyed in Guangzhou, Huizhou, Zhaoqing, and Jiangmen, the mean soil TP concentration of 1.87, 1.23, 1.29, and 1.42 g P kg^-1^ was recorded, respectively (Fig 2A). Overall, soil TP concentrations in the research area ranged from 0.39 to 3.92 g P kg^-1^, with an average of 1.55 g P kg^-1^ (Fig 2A). In the majority of soil samples (73.2% of total samples), TP concentration ranged from 1.01 to 2.0 g P kg^-1^ (Fig 2B), which corresponds to a medium to very rich status, as defined by the soil nutrient classification standards from the Second National Soil Census in China [39]. Notably, 24.2% of the total soil samples were characterized by TP concentrations exceeding 2.0 g P kg^-1^ (Fig 2B). The AP concentration of soil samples from Guangzhou, Huizhou, Zhaoqing, and Jiangmen was 147.6, 100.92, 102.04, and 119.15 mg P kg^-1^ (Fig 3A), respectively. Within the research area, a total of 93.7% of the soil samples were found to have an AP concentration higher than 40 mg P kg^-1^ (Fig 3B), which is defined as the threshold of a very rich status for AP based on the soil nutrient classification standards (S1 Table) [39]. Overall, soil AP concentration in the research area ranged from 13.8 to 314 mg P kg^-1^, with an average value of 124.49 mg P kg^-1^. The ratio of soil AP to TP is defined as the phosphorus activation coefficient (PAC) [40], and in the present study, the mean PAC value of 8.33%, 8.69%, 8.27%, and 8.33% was obtained, with corresponding coefficients of variation (CV) of 44.8%, 32.5%, 40.4%, and 37.1%, for soils collected in Guangzhou, Huizhou, Zhaoqing, and Jiangmeng, respectively (Table 2). At the regional scale (the entire research area), PAC values ranged from 1.58 to 19.89%, with an average of 8.39% (Table 2).

**Table 2:** Phosphorus activation coefficient in soil from different sampling sites (n = 190).

Parameters	Guangzhou	Huizhou	Zhaoqing	Jiangmen	Research area
Average (%)	8.33	8.69	8.27	8.33	8.39
Range (%)	1.58–19.89	4.13–15.47	3.74–18.8	2.54–15.58	1.58–19.89
C.V. (%)	44.8	32.5	40.4	37.1	40.0

C.V., Coefficient of variation; Research area, includes Guangzhou, Huizhou, Zhaoqing and Jiangmen.

Based on data provided by the Second National Soil Census in China in 1980, soil TP and AP levels in the farmlands of Guangdong Province were 0.087% ± 0.010% (equivalent to 0.87 g P kg^-1^) and 14.6±3.4 mg P kg^-1^ [31], respectively. Our findings in the present study revealed that there have been significant increases in both soil TP and AP concentrations in the period from 1980 to 2019, which were estimated to have increased by 78% and 753%, respectively, over the past four decades. Moreover, within this region, soil TP and AP accumulation rates of 17.4 mg P kg^-1^ year^-1^ and 2.82 mg P kg^-1^ year^-1^ was observed, respectively.

### 3.2 Soil properties

The major properties of the 11 soils selected for isotherm sorption measurements are displayed in Table 3. Most of the soils were slightly acidic, with pH values ranging from 5.04 to 6.46; the exception being soil S8 with a pH of 7.23. The soils are generally sandy loams to silty clay loams, with silt contents ranging from 28.8% to 52.4% (mean: 44.69%) and a clay content ranging from 13.8% to 29.4% (average: 21.78%) (Table 3). The mean concentration of organic matter (OM), TP and AP was 21.19 g kg^-1^, 1.58 g kg^-1^, and 126.59 mg kg^-1^, respectively (Table 3). Cation exchange capacity (CEC) averaged 9.16 cmol kg^-1^, ranging from 4.71 to 17.0 cmol kg^-1^, with values for a majority of the soils being lower than 10 cmol kg^-1^. The concentrations of elements extracted using DCB ranged from 2.33 to 5.00 g kg^-1^ for Al_d_, 11.16 to 30.72 g kg^-1^ for Fe_d_, and 47.24 to 591.47 mg kg^-1^ for Mn_d_; whereas, the concentrations of amorphous Al, Mn, and Fe oxides (Al_o,_ Mn_o_ and Fe_o_), extracted with ammonium oxalate, ranged between 1.73 and 3.23 g kg^-1^, 41.12 and 586.53 mg kg^-1^, and 2.27 and 9.68 g kg^-1^, respectively.

**Table 3:** Physico-chemical properties of the eleven soils from different vegetables fields (n = 11).

Soil samples*	pH	Sand	Silt	Clay	OM	TP	AP	CEC	Al_d_	Mn_d_	Fe_d_	Al_o_	Mn_o_	Fe_o_
		%	%	%	g/kg	g/kg	mg/kg	cmol/kg	g/kg	mg/kg	g/kg	g/kg	mg/kg	g/kg
S1	5.92	20.6	52.4	27.0	26.38	1.64	109.80	13.12	3.61	295.75	12.58	3.06	197.86	3.73
S2	5.05	32.6	48.4	19.0	37.84	2.59	228.71	17.00	4.64	505.71	30.72	3.23	328.01	9.68
S3	5.83	46.2	28.8	25.0	20.57	0.87	13.80	4.71	3.52	84.43	22.33	1.73	67.94	2.27
S4	5.61	26.6	48.4	25.0	20.53	1.42	101.27	13.86	2.72	587.90	15.90	2.11	586.53	7.83
S5	5.04	19.2	51.4	29.4	17.36	1.75	179.35	7.80	5.00	64.92	21.45	2.31	60.21	4.87
S6	6.46	36.6	41.2	22.2	14.26	1.35	119.67	7.64	3.44	52.85	13.93	1.91	44.29	2.58
S7	6.14	39.0	41.6	19.4	11.83	0.96	74.35	6.92	2.33	591.47	11.16	1.89	523.74	4.04
S8	7.23	40.2	46.0	13.8	27.38	2.07	167.04	7.60	2.68	143.20	20.88	1.79	103.57	5.04
S9	5.87	37.6	46.6	15.8	18.55	1.33	106.98	6.12	3.76	132.71	20.53	2.05	100.86	4.09
S10	5.12	42.0	38.2	19.8	25.22	1.86	179.74	8.15	2.63	47.24	14.77	2.03	41.12	5.57
S11	6.04	28.2	48.6	23.2	13.21	1.57	111.75	7.89	4.28	243.15	28.85	2.42	176.93	4.08
Minimum	5.04	19.20	28.80	13.80	11.83	0.87	13.80	4.71	2.33	47.24	11.16	1.73	41.12	2.27
Maximum	7.23	46.20	52.40	29.40	37.84	2.59	228.71	17.00	5.00	591.47	30.72	3.23	586.53	9.68
Average	5.85	33.53	44.69	21.78	21.19	1.58	126.59	9.16	3.51	249.94	19.37	2.23	202.82	4.89
C.V. (%)	11.20	26.38	15.32	21.78	35.95	30.96	46.53	41.20	24.90	86.29	33.12	22.32	95.69	44.64

* Information of eleven soils see Table 1. TP, total phosphorus; AP, available phosphorus; OC, organic carbon, obtained from organic matter content (OM) divided by 1.724. CEC, cation exchange capacity; Al_d_, free aluminium oxides; Mn_d_, free manganese oxide; Fe_d_, free iron oxide; Alo, amorphous aluminum oxides; Al_o_, free aluminum oxide; Fe_o_, amorphous iron oxide. C.V., Coefficient of variation.

According to the soil nutrient classification standards from the Second National Soil census in China (S1 Table) [39], soil OM levels are in the deficient to rich range and TP levels are in the medium to very rich range. However, most of soil AP concentrations are in the very rich status with an exception in sample S3, for which AP value is in medium level.

### 3.3 P adsorption

**Fig 4:**
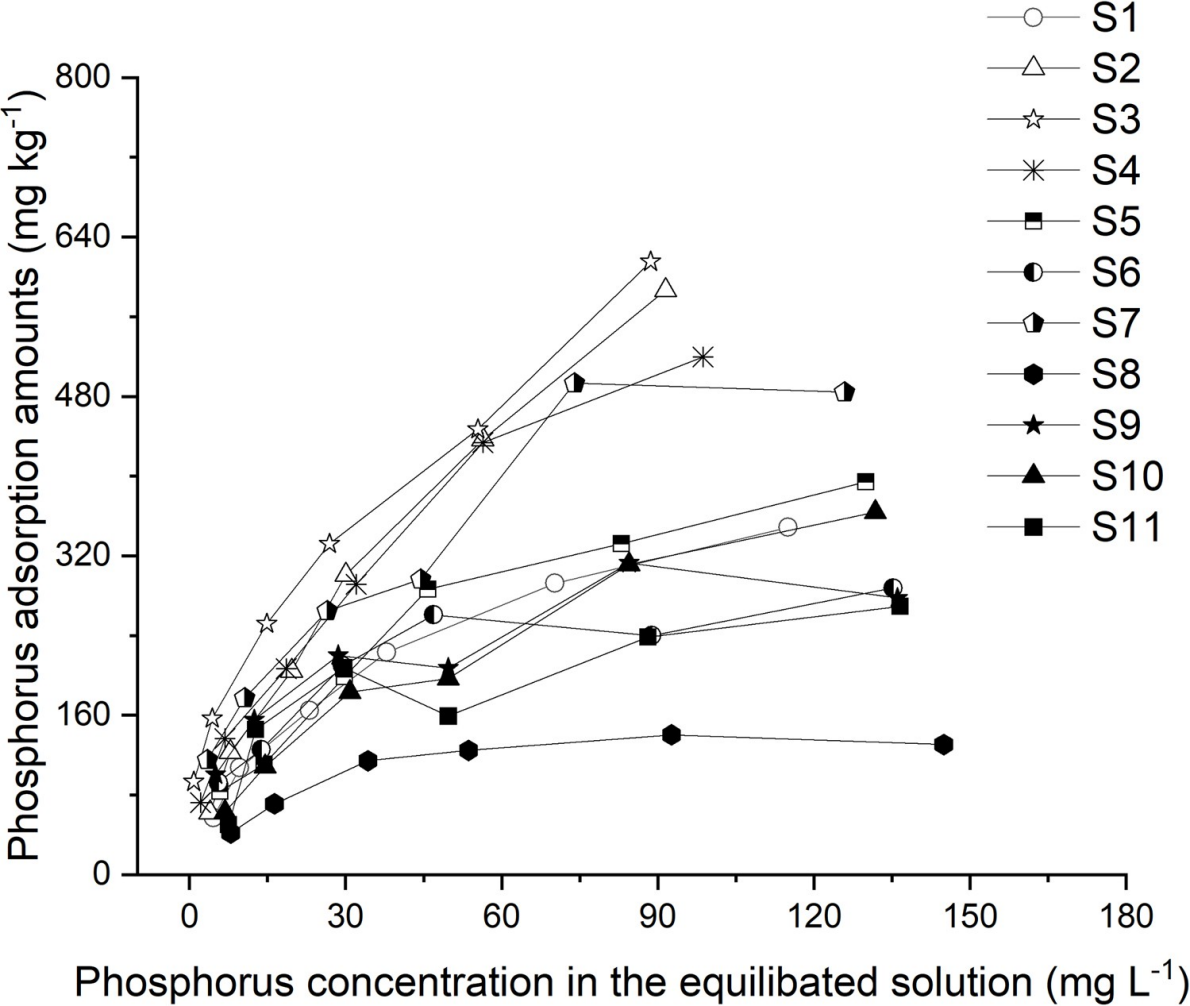
Phosphorus (P) adsorption isotherms in soil from different fields (n = 11)*. * Information of eleven soils see Table 1.

In all assessed soils, P sorption increased non-linearly with increasing P concentrations (Fig 4). However, the percentage of adsorbed P (the ratio of adsorbed P to added P) decreased as the concentration of P increased. For soils with P application rates of 10 and 150 mg L^-1^, the adsorbed fraction of 20.89%–57.67% and 4.35%–20.51% was recorded, respectively. It was also found that for all soils, the Langmuir model provided a satisfactory description of P sorption (R^2^ = 0.92–0.99) (Table 4). For the current soils, the Q_m_ values ranged from 142.86 to 909.09 mg kg^-1^, with a mean value of 488.38 mg kg^-1^ (Table 4). Compared to the values obtained for the P adsorption capacity in this area in 1980, which was a mean value of 581.73 mg kg^-1^ with a range of 75 to 738 mg P kg^-1^ [41], the values for current soils were slightly lower. Furthermore, for soils in the research area, we obtained a mean k value of 0.048 L mg^-1^, ranging from 0.017 to 0.123 L mg^-1^; a mean MBC value of 18.82 L kg^-1^, ranging from 9.15 to 37.33 L kg^-1^; and a mean EPC_0_ value of 0.030 mg L^-1^, with a wide variation of 0.001 to 0.085 mg L^-1^ (Table 4).

**Table 4:** Isotherm parameters of the Langmuir equation for soil P adsorption (n = 11).

Soil samples	k (L mg^-1^)	Q_m_ (mg kg^-1^)	R^2^	MBC (L kg^-1^)	EPC_0_ (mg L^-1^)
S1	0.030	434.78	0.99	13.04	0.001
S2	0.017	909.09	0.96	15.45	0.046
S3	0.056	666.67	0.92	37.33	0.004
S4	0.033	666.67	0.94	22.00	0.015
S5	0.023	526.32	0.97	12.11	0.028
S6	0.064	312.5	0.98	20.00	0.022
S7	0.039	588.24	0.94	22.94	0.015
S8	0.123	142.86	0.99	17.57	0.065
S9	0.079	312.5	0.97	24.69	0.008
S10	0.018	500	0.97	9.15	0.085
S11	0.041	312.5	0.95	12.75	0.039
Minimum	0.017	142.86	0.92	9.15	0.001
Maximum	0.123	909.09	0.99	37.33	0.085
Average	0.048	488.38	0.96	18.82	0.030
C.V. (%)	66.74	44.26	2.32	42.08	89.40

*Information of eleven soils see Table 1. Q_m_, phosphorus sorption maximum; EPC0, the equilibrium phosphorus concentration at zero adsorption; MBC, the maximum adsorption buffering capacity; k, a constant related to the binding energy. C.V., Coefficient of variation.

### 3.4 Correlations between soil P contents, P adsorption, and soil properties

**Fig 5:**
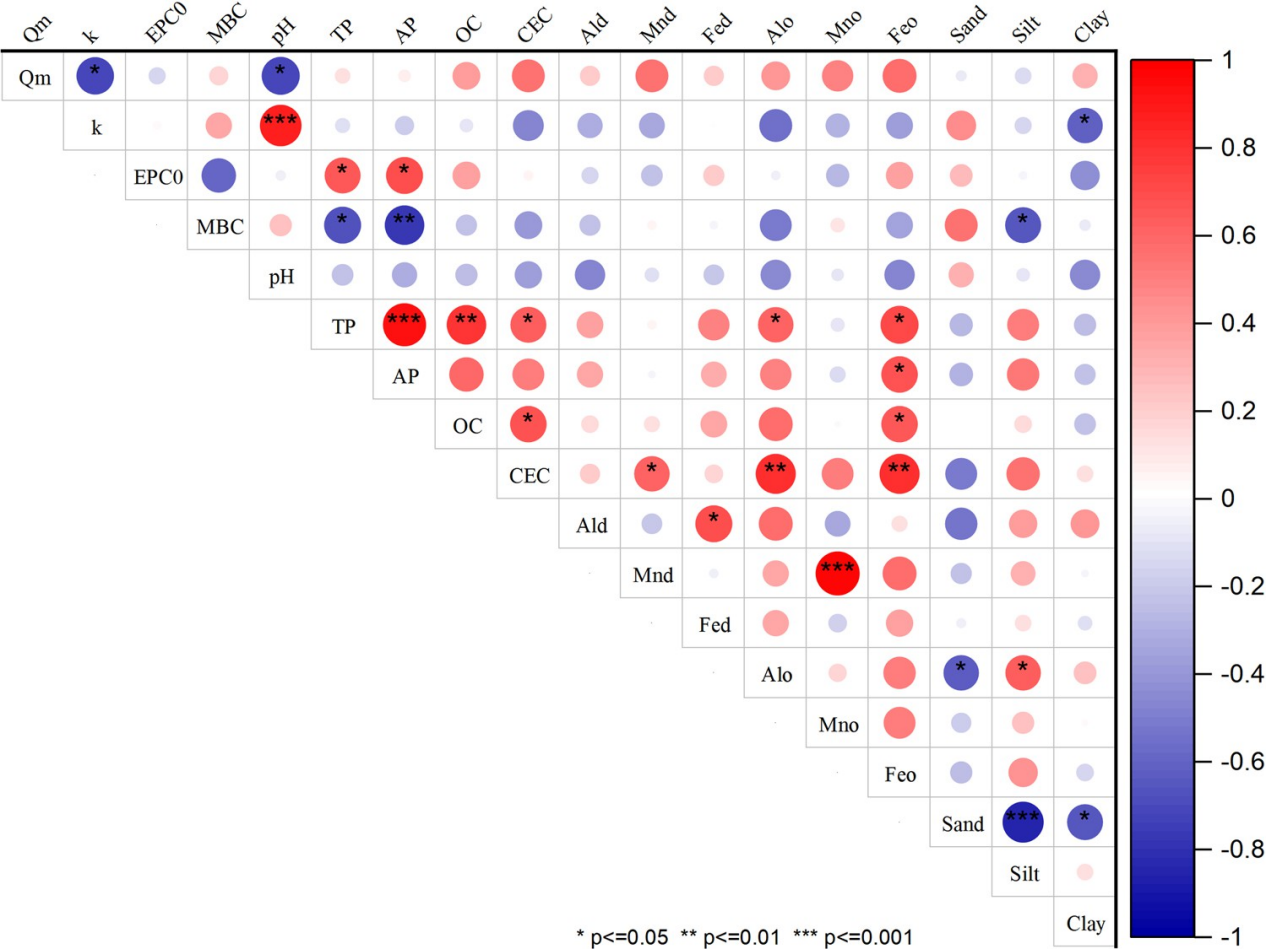
Correlation matrix between soil properties (n = 11)*. * Information of eleven soils see Table 1. Q_m_, phosphorus sorption maximum; k, a constant related to the binding energy; EPC0, the equilibrium phosphorus concentration at zero adsorption; MBC, the maximum adsorption buffering capacity; TP, total phosphorus; AP, available phosphorus; OC, organic carbon, obtained from organic matter content (OM) divided by 1.724. CEC, cation exchange capacity; Al_d_, free aluminium oxides; Mn_d_, free manganese oxide; Fe_d_, free iron oxide; Alo, amorphous aluminum oxides; Al_o_, free aluminum oxide; Fe_o_, amorphous iron oxide.

Pearson’s correlation analysis revealed that TP was significantly correlated with the contents of organic carbon (OC, obtained from the organic matter content divided by 1.724), amorphous Al (Al_o_), Fe (Fe_o_), and CEC (Fig 5). A significant correlation between AP and Fe_o_ was also observed. No significant correlations were detected between soil P concentrations and soil texture (sand, silt, and clay contents) (Fig 5).

EPC_0_ was found to be positively correlated with soil P concentration (TP and AP) (*p* < 0.05), but we detected a negative correlation between MBC and P concentration (*p* < 0.05, *p*< 0.01) (Fig 5). Moreover, it was observed that the soil silt content was significantly correlated with MBC (Table 4), and a positive correlation between soil pH and the k value (*p*< 0.01), as well as a negative correlation between soil clay content and k value (*p*< 0.05) were observed (Fig 5). However, Q_m_ was only significantly correlated with soil pH (*p*<0.05) (Fig 5). In addition, on the basis of stepwise multiple regression analysis, a two-term model based on pH, Mn_d_, and silt that explained 87% of the variation in Q_m_, whereas pH and Mn_d_ together accounted for 74% of the variation (Table 5).

**Table 5:** Multiple regression formulae describing the relationship between soil properties and P sorption maxima for 11 vegetable-cultivated soils*.

Response	Independent variables	Equation	Statistics
Q_m_	pH, Mn_d_, silt	y = 2207.482–227.87pH+0.586Mn_d_-11.936Silt	R^2^ = 0.87 p<0.01
Q_m_	pH, Mn_d_	y = 1658.69–220.41pH+0.473Mn_d_	R^2^ = 0.74 p<0.01

*Information of eleven soils see Table 1. Mn_d_, free manganese oxide.

## 4. Discussion

In the area surveyed in this study, excessive accumulation of soil P and a concomitant reduction in P adsorption capacity have been recorded over the past four decades. Soil physicochemical properties have been found to have significant effects on the soil P pool, as well as the availability of P via soil P adsorption. Accordingly, factors such as anthropic activities and soil-associated intrinsic qualities influence the soil properties, as discussed below.

The soil in the research area is a latosolic red soil developed primarily from the underlying parent material of granite and sandy shale. It is characterized by a low pH (~5.0) and low base cations (0.3–4.91 mEq/100 g soil), as well as a low SiO_2_ to Al_2_O_3_ ratio (1.7–2.3), and high concentrations of Fe_2_O_3_ (5.06–17.49%) and Al_2_O_3_ (28.17–37.76%) [31], indicating a strong phosphate-fixing capacity. In this study, the significant correlations between soil TP and amorphous Fe/Al, as well as between AP and amorphous Fe (Fig 5) were observed. It was suggested that Fe/Al oxides contribute to the accumulation of the soil P pool, on account of their high specific surface areas and reactivity [42]. This can be considered an intrinsic factor promoting soil P accumulation. Moreover, a large number of studies have investigated the role of chemical fertilizers on soil P accumulation, based on a range of different field experiments [5, 43–48]. Notably, in the present study, it was found that since 1980, there have been significant increases of 78% and 753% in soil TP and AP, respectively, which can largely be attributed to a massive increase (479%) in the input of chemical phosphate fertilizer over the past four decades [6]. In this regard, the soils assessed in the present study were collected from perennial vegetable-cultivated fields, in which annual chemical nutrient *(*N+P_2_O_5_+K_2_O) inputs have reached to the level of 1639.5 kg hm^-2^, which is 65% higher than the average input in provincial-scale farmland systems [49]. In addition, the annual rate of AP accumulation in soil (i.e., 2.64 mg P kg^-1^) in the research area was higher than that at the national scale (i.e., 0.64 mg P kg^-1^) that was reported by Li et al. (2011) [5]. The difference between these two figures reflects differences in the properties of soils receiving chemical fertilizer input [6]. Therefore, we can identify anthropic activities, primarily chemical phosphate fertilizer application, as vital exogenous factors that contribute to the current soil P surplus observed in the research area. PAC is an important indicator of soil fertility, representing variations in (and degrees of difficulty of) the transformations between TP and AP [40]. According to Wang et al. (2014) [50], TP is not readily converted to AP when PAC is less than 2.0%. On average, soil PAC in the research area was found to be higher than 2.0%, indicating that soil TP represents a potential AP pool for providing P nutrients for vegetable growth.

The adsorption of P by clay minerals in soil is a physicochemical process that influences both the availability and accumulation of P. In this study, a greater proportion of added P was adsorbed at low P concentrations, indicating that chemical adsorption dominates the adsorption processes when percentage P contents are relatively low where ion exchange and ligand exchange are considered to be the main mechanisms contributing to the high adsorption rate [51, 52]. Similar results have been reported in previous studies [53–55]. To describe the P adsorption process, the Langmuir equation was used to calculate the parameters of EPC_0_, k, MBC, and Q_m_ to determine the P availability and adsorption capacity of soil [56]. Specifically, EPC_0_ was used to evaluate the P interactions between soils and soil solutions [57]. Our findings indicated that the assessed soils have a strong ability to retain P, as the values obtained for EPC_0_ (0.001 to 0.085 mg L^-1^; Table 4) were lower than those reported in previous studies [17, 57, 58]. In the case of P-binding strength, the k value describes the affinity of soil for P [59]. According to Castro and Torrent (1998) [60], a k value lower than 0.4 L mg^-1^ indicates that adsorption, rather than precipitation, is the primary process whereby P is removed from soil solutions. Thus, it was indicated that the removal of P from the soil solutions examined in the present study occurred via adsorption. MBC can be used to assess the supply and immobilization of soil P. The soil MBC values obtained in this study were found to be similar to those previously described for a red soil [58], although they are lower than those of a black soil [51]. To a large extent, this disparity among soil types simply reflected the fact that the properties of the soil examined in the present study are more similar to those of the red soil than to those of the black soil. In particular, the negative correlations between soil P (TP and AP) concentration and MBC, and the positive correlation between soil P concentration and EPC_0_ (Fig 5) indicated that soils with high levels of available P tend to have low MBC values, as most of their reactive sites are saturated with phosphates and orthophosphate ions. This is consistent with the findings reported by Sun et al. (2020) [55].

Q_m_ is considered an indicator of the capacity of soil to interact with phosphate, which to a certain extent governs soil P availability [18]. In this regard, previous studies have reported that long-term fertilization can modify the P adsorption capacity of soil [61–63]. In the present study, it was found that the fertilization of soil over the past four decades has had the effect of reducing the P adsorption capacity of soils in the research area, and we accordingly speculate that continuous application of fertilizers has contributed to a reduction in available exchange sites on the soil surface layer. Our findings are consistent with those reported previously by Guo et al. (2008) [37], Sharma et al. (1995) [64], and Abboud et al. (2018) [65]. According to the previous reports, it is considered that an increase of total P, organic C or pH in soil is probably to induce a decrease in P adsorption [28, 66, 67]. In this study, the significantly negative correlation between Q_m_ and soil pH emphasizes the importance of pH in P adsorption and implied that P adsorption decreases as the negative charge density in soil colloids increases with the increase of soil pH [68, 69]. This can be attributed to the competition between hydroxyl ions (OH^-^) and phosphate ions for specific sorption sites [62, 70]. However, Zhang et al. (2005) [71] found soil pH to be uncorrelated with the maximum P sorption, whereas Agbenin (1996) [72] observed an increasing trend in P sorption with increasing pH in some savannah soils. These conflicting findings can probably be attributed to differences in soil properties, such as soil pH, soil type, and clay mineral constituents, which can potentially affect the adsorption capacity of soil.

Compared to the elements of Fe and Al, Mn tends to receive relatively less attention with respect to its interaction with P in soil. In this study, however, it was observed that Mn_d_, rather than Fe or Al oxides, contributed significantly to explaining the variations in soil Q_m_. Similarly, Jugsujinda et al. (1995) [73] found that P adsorption in acid sulfate soils was significantly affected by exchangeable Mn, which are mainly present in this type of soil [74]. Consistently, Liao and Lu (1996) [75] observed a stronger PO_4_^3-^ sorption capacity of manganese hydroxide than that of either iron hydroxide or aluminum hydroxide, within the first 20 days under incubation conditions. At present, however, it is difficult to explain these anomalous findings, owing to the comparatively limited information relating to the interaction between Mn and P sorption. In addition to pH and Mn_d_, soil texture is also believed to contribute to the observed variations in Q_m_, albeit to a lesser extent. In particular, it was found that silt was more closely associated with Q_m_ than the clay content of soil, which was consistent with the findings reported by Nwoke et al. (2003) [70] and Zhang et al. (2019) [76].

## 5. Conclusions

The results obtained in this study have revealed that over the past four decades, a large accumulation of phosphorus, accompanied by reductions in soil P sorption capacity, have occurred in the intensively managed agricultural soils (perennially cultivated vegetable field soils) of southern China. Both soil properties and anthropic activities contributed to this augmentation of the soil P pool, with long-term fertilization being considered the main factor driving P accumulation. This accumulation was observed to be negatively correlated with the soil’s maximum adsorption buffering capacity and positively affected the P-retention capacity of soil (i.e., EPC_0_). Soil properties, i.e. pH, manganese oxide concentrations, and percentage of silt content all contributed to the variation of soil P sorption maximum. Collectively, the findings of this study provide a basis for designing suitable P management strategies for the sustainable utilization of agricultural soils in Guangdong Province, South China.

## Supporting information

S1 Table Classification of soil organic matter and phosphorus contents. (XLS)

S2 Table Original data for Fig 2A concentrations of total phosphorus in studied soils. (XLS)

S3 Table Original data for Fig 2B frequency distribution of total phosphorus in studied soils. (XLS)

S4 Table Original data for Fig 3A concentrations of available phosphorus in studied soils. (XLS)

S5 Table Original data for Fig 3B frequency distribution of available phosphorus in studied soils. (XLS)

S6 Table Original data for Table 2 phosphorus activation coefficient in soil from different sampling sites (n = 190). (XLS)

S7 Table Original data for Fig 4 phosphorus (P) adsorption isotherms in soil from different fields (n = 11). (XLS)
